# Brain Circuits Involved in the Development of Chronic Musculoskeletal Pain: Evidence From Non-invasive Brain Stimulation

**DOI:** 10.3389/fneur.2021.732034

**Published:** 2021-08-31

**Authors:** Mina Kandić, Vera Moliadze, Jamila Andoh, Herta Flor, Frauke Nees

**Affiliations:** ^1^Institute of Cognitive and Clinical Neuroscience, Central Institute of Mental Health, Medical Faculty Mannheim, Heidelberg University, Mannheim, Germany; ^2^Institute of Medical Psychology and Medical Sociology, University Hospital Schleswig-Holstein, Kiel University, Kiel, Germany; ^3^Department of Psychiatry and Psychotherapy, Medical Faculty Mannheim, Central Institute of Mental Health, University of Heidelberg, Mannheim, Germany

**Keywords:** non-invasive brain stimulation, transcranial magnetic stimulation, transcranial alternating current stimulation, transcranial direct current stimulation, development of chronic musculoskeletal pain, brain mechanisms

## Abstract

It has been well-documented that the brain changes in states of chronic pain. Less is known about changes in the brain that predict the transition from acute to chronic pain. Evidence from neuroimaging studies suggests a shift from brain regions involved in nociceptive processing to corticostriatal brain regions that are instrumental in the processing of reward and emotional learning in the transition to the chronic state. In addition, dysfunction in descending pain modulatory circuits encompassing the periaqueductal gray and the rostral anterior cingulate cortex may also be a key risk factor for pain chronicity. Although longitudinal imaging studies have revealed potential predictors of pain chronicity, their causal role has not yet been determined. Here we review evidence from studies that involve non-invasive brain stimulation to elucidate to what extent they may help to elucidate the brain circuits involved in pain chronicity. Especially, we focus on studies using non-invasive brain stimulation techniques [e.g., transcranial magnetic stimulation (TMS), particularly its repetitive form (rTMS), transcranial alternating current stimulation (tACS), and transcranial direct current stimulation (tDCS)] in the context of musculoskeletal pain chronicity. We focus on the role of the motor cortex because of its known contribution to sensory components of pain via thalamic inhibition, and the role of the dorsolateral prefrontal cortex because of its role on cognitive and affective processing of pain. We will also discuss findings from studies using experimentally induced prolonged pain and studies implicating the DLPFC, which may shed light on the earliest transition phase to chronicity. We propose that combined brain stimulation and imaging studies might further advance mechanistic models of the chronicity process and involved brain circuits. Implications and challenges for translating the research on mechanistic models of the development of chronic pain to clinical practice will also be addressed.

## Introduction

Chronic musculoskeletal pain is defined as a persisting or reoccurring pain that originates in musculoskeletal structure (1). In the new ICD-11 classification it is listed under the chronic primary pain category where it is recognized as a “disease in its own right” that cannot be explained by another disease (2).

Living with chronic musculoskeletal pain is a great burden to an individual experiencing pain, along with large-scale implications for society including enormous medical annual costs worldwide, the occurrence of sick leave, and work disability (3). Despite these high individual and societal costs, efforts to effectively treat chronic pain have been met with moderate success and in many patients, chronic pain remains untreated or poorly treated (4). The prevention of the transition from acute to chronic pain is therefore an important goal. The mechanisms of this transition remain, however, poorly understood (4).

Research has revealed that the brain in chronic and acute pain stage differs (5). However, the relationship between changes in a certain brain circuit and pain is never one-dimensional, since these alterations in the brain can relate to other factors such as medication intake and affective comorbidity (6–8). Since it has been shown that circuits that subserve emotional, learning, reward, and memory processes are key factors in the development of chronicity, these mechanisms themselves could be driving forces or catalysts of the transition (9–11). Moreover, genetic, and epigenetic factors (12), physiological and psychosocial expressions of stress (13, 14), have also been implicated in the development of chronic musculoskeletal pain. This stresses the importance of considering these factors when modeling and investigating brain-pain relationship. A mechanistic model of pain therefore acknowledges several key elements and their interaction in chronic pain pathogenesis and maintenance (15).

Multiple emotional and cognitive factors impact on the experience of pain, thus brain circuits involved in the processing of emotion might play an important role in the development of chronic pain (16, 17). Evidence from longitudinal imaging studies suggests a shift from brain regions involved in nociceptive processes toward brain areas supporting emotion, motivation, and memory processes when acute musculoskeletal pain persists (5, 18). Such findings are an important step toward unraveling neural changes associated with chronic pain.

Although neuroimaging studies allowed to further advance our knowledge about plastic changes related to pain chronicity, they cannot provide causal relationships between them. In this context, non-invasive brain stimulation (NIBS) methods have been used to modulate cortical excitability in specific brain areas, in order to show a direct relationship between brain and behavior. NIBS allows a step further into the understanding on the mechanisms of pain, acute and chronic, and can be applied on both healthy participants and chronic pain patients (19).

**Figure 1:**
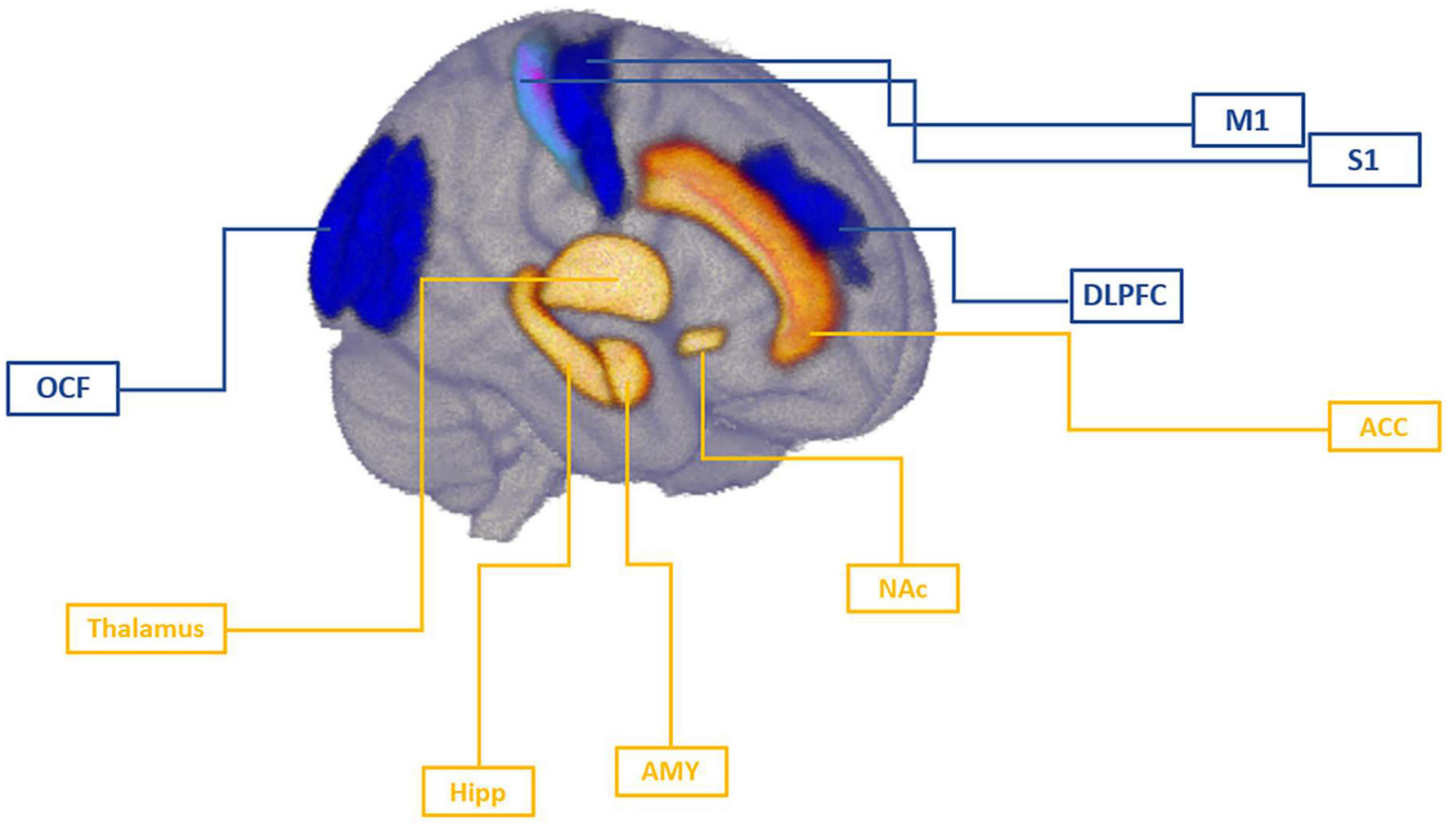
Brain targets involved in the mechanism of pain chronicity. Cortical targets of NIBS studies were primary motor cortex (M1), primary somatosensory cortex (S1), dorsolateral prefrontal cortex (DLPFC), and occipital field (OCF). Imaging studies have identified thalamus, hippocampus (Hipp), amygdala (AMY), and nucleus accumbens (NAc) as key subcortical regions implicated in pain chronicity. Anterior cingulate cortex (ACC) has been identified as an important relay in the medial pain pathway integrating sensory, attentional, and motivational components of pain that can also been targeted via cortical stimulation due to its interconnections with these targets. In tonic, acute pain stage, motor cortex, as well as somatosensory regions may undergo rapid changes as response to peripheral insult, and the magnitude of this response might be shaped by pre-existing individual differences within these regions. As demonstrated in NIBS studies, DLPFC regulates top-down inhibition of pain independently of motor cortex activation, possibly modulating sensory component in an early stage of pain development. As pain develops to the chronic stage, cognitive aspects of pain become more important mechanism and prefrontal regions may regulate the affective component of pain via their influence on cingulate and limbic circuits.

In this review, we will present an overview of the available NIBS studies with respect to pain development and discuss these studies in the context of a mechanistic understanding of pain chronicity. We further review to what extent NIBS studies offer additional targets on brain circuits (see Figure 1) involved in transition from acute to chronic pain. Lastly, we suggest future directions for NIBS research and discuss implications for the clinical practice.

## Neural Circuits Involved in Chronic Musculoskeletal Pain: Neuroimaging Evidence

Neuroimaging studies in chronic pain patients have revealed altered structure and function of the brain in chronic pain. Studies in fibromyalgia, chronic tension-type headache, and chronic back pain patients have reported structural and functional changes in regions not typically involved in nociceptive processing, such as limbic and prefrontal cortices (16, 20, 21). Furthermore, the default mode network (DMN), which is active in the absence of any task to maintain resting brain activity and is deactivated during task-based fmri (functional magnetic resonance imaging), showed persistent increased activity rather than deactivation in chronic pain patients (22) and abnormal functional connectivity with other brain regions at rest (23, 24) that was associated with the duration of chronic pain (24, 25).

In addition, the brain of chronic pain patients might differentially process acute and chronic pain, with the prefrontal cortex being a key region for this dissociation (16). Moreover, the prefrontal cortex is also believed to constitute one of the key regions in descending inhibitory pathways (26), and this pathway has also been found to be impaired in many chronic pain conditions, including chronic musculoskeletal pain (27, 28). Neuroimaging evidence documented distinct neural patterns for tonic and chronic pain in comparison to experimental phasic pain, with somatomotor, frontoparietal, and dorsal attention networks emerging as key circuits (29).

From the few available longitudinal data, it seems that similar networks are involved in the acute to chronic transition process, and that particularly corticostriatal circuits play an important role in pain chronicity. In a sample of subacute back pain patients followed over 1 year, increased functional connectivity between the medial prefrontal cortex (mPFC) and the nucleus accumbens (NAc) (18), and decreased hippocampal-medial prefrontal cortex functional connectivity during spontaneous pain were found predictive for the transition to pain chronicity (30). In addition, volumes of amygdala, hippocampus (31), and NAc (32) could predict chronic pain development. In further support of these results, denser white matter corticolimbic connections, specifically between mPFC and NAc, predicted the shift to the chronic state after a 1 year period (33).

## Non-invasive Brain Stimulation: Basic Priniciples and Findings in Chronic Musculoskeletal Pain

Two methods of non-invasive transcranial brain stimulation have dominated recent decades: transcranial magnetic stimulation (TMS), which activates axons through short-pulsed stimulation and leads thereby to new action potentials; and transcranial electric stimulation (tES), most used transcranial direct current stimulation (tDCS), which can be used to manipulate the membrane potential of neurons and modulate spontaneous firing rates, but which by itself is not sufficient to discharge resting neurons or axons (34).

### Transcranial Magnetic Stimulation

TMS operates on the electromagnetic induction principle (35). The device unit typically encompasses a capacitor that accumulates and discharges a rapidly changing current of high voltage through the transducing coil placed to the subject's head. This sequentially creates a powerful (2–3 T) and brief (100–200 μs) magnetic field in the wires of the coil, inducing in turn an electrical field perpendicular to the coil surface in the neural tissue beneath the coil (36). The current induced at the neuronal layer is attenuated through the cranium and extracerebral tissues, yet it can exert enough strength to act in a suprathreshold manner and elicit an action potential (37). TMS primarily affects neurons in superficial areas directly below the coil, where the intensity of the current decays with the distance away from the coil (38). Different coils produce slightly different electrical field strengths and spreads, but all follow a depth-focality trade-off (39). Apart from local effects, stimulation can induce distant effects via propagation to interconnected regions belonging to the same neural network (40). Spread of the current is dependent on individual and tissue properties, which, however, cannot be controlled, and stimulation parameters that can be selected such as geometry of the coil, pulse waveform, intensity, frequency, and number of delivered pulses (41, 42).

A range of combinations of possible parameters constitutes the stimulation patterns that serve different purposes. For example, single pulses are applied in studies that investigate functioning of brain regions, while paired pulse regimes can be used to explore inhibitory or excitatory intracortical networks or connectivity of two cortical regions via conduction time that two successive pulses induce between them (43). When delivered in repetitive stimulation trains, the TMS regime is termed *repetitive transcranial magnetic stimulation* with low repetition rates under or at about 1 Hz decreasing excitation, whereas high frequencies of ≥5 Hz are generally believed to increase excitability of stimulated region (36). Due to the short interstimulus period, effects of rTMS sum up and can modulate neural activity beyond the stimulation period, thereby promoting neuronal plasticity (44). It is assumed that rTMS after-effects are based on long-term potentiation (LTP) and long-term depression-like (LTD)-like mechanisms of synaptic plasticity (36, 37). Recently, neurostimulation research has become interested in theta-burst stimulation (TBS), a modification of high-frequency rTMS. There is evidence that TBS produces even more robust changes in cortical excitability than those observed in the conventional rTMS protocols. TBS typically consists of bursts of three pulses at 30 Hz or 50 Hz, repeated five times per second with 600 pulses in total (i.e., at theta frequency). There are two different paradigms: intermittent TBS (iTBS) and continuous TBS (cTBS). While iTBS facilitates CE, cTBS attenuates it (45–49). The advantage of TBS as compared to low and high frequency rTMS is that by using a similar number of pulses but considerably shorter duration and lower intensity of stimulation, experimental time is reduced without jeopardizing effect strength.

Using TMS, the brain can be briefly activated or briefly inhibited. Applications were first in the motor system and have now been used to map sensory processes and cognitive function. When TMS is delivered onto the primary motor cortex, it has the capacity to initiate descending volleys from pyramidal axons to spinal motor neurons, as demonstrated by epidural recordings in anesthetized humans (50). When the target of stimulation is a region subserving higher cortical function, TMS can interfere with neuronal firing and intercommunication within that region, which has been termed “virtual lesion” and reflects a momentary disruption of ongoing neuronal activity (51). Corresponding effects in the cognitive and/or behavioral domain can be measured through specific tasks, and enable to establish brain-behavior relationships. Such change in behavior can be observed online, i.e., being the product of concurrent stimulation, or offline, i.e., immediately after or up to an hour after the stimulation period, called after-effects. These effects emerge as a result of repeated depolarization events that temporally change neuronal firing (40).

### Transcranial Direct and Alternating Current Stimulation

Transcranial current stimulation employs electric current through two or more surface electrodes attached directly to scalp and connected to the battery-driven stimulator (52). Unlike strong magnetically induced electric field in TMS, electrical current produced by TES (Transcranial electrical stimulation) is of weaker potential (53). This leaves the neural tissue excited below the necessary threshold to produce an action potential, but sufficient to modulate the firing of neurons in case of upcoming neural input (54). In general, direct current has been shown to influence a range of different neurotransmitters [for review, see (55)], while the long-lasting effects thought to induce plasticity have been attributed to the modulation of the N-Methyl-D-aspartate (NMDA) receptors and GABA modulation has a gating function on respective plasticity (56, 57).

The most common bipolar montage comprises of anode and cathode electrodes, producing polarity-specific modulation effects. Following the simplified assumption, in tDCS, a constant current flows between the electrodes, with anodal stimulation increasing cortical excitability and cathodal stimulation decreasing it (58). Similarly to TMS, the modulatory effects induced by tDCS depend on the choice of electric current intensity, waveform, and position and size of electrodes (59). Computational modeling studies suggested that current flow is mostly focused under the stimulated electrode (53), although human imaging studies showed that tDCS could even modulate spinal network excitability (60), which is in line with animal studies showing spread of tDCS-related effects to subcortical networks (61). Novel approaches emerged to improve spatial targeting, such as high definition transcranial current stimulation (HD-tCS) (62) or network-targeted multichannel stimulation (net-tCS) (63, 64) that make use of multiple electrodes improving focality.

Transcranial alternating current stimulation (tACS) occupies intermediate positions, in the physical sense, between pulsed rTMS and continuous tDCS. In tACS, the electrical current alternates between electrodes, usually in a sinusoidal form (65). The exact mechanisms by which tACS modulates brain activity are still not fully understood, five common explanations for direct, modulatory “online” effects include stochastic resonance and rhythm resonance, temporal biasing of spikes, network entrainment and imposed patterns (66). These mechanisms are assumed to affect activity in larger networks in the brain. Contrary to these suggested direct online mechanisms of electrical stimulation, the after-effects of tACS likely depend on the induction of neural plasticity (67). When brain activity is aimed to be modulated in a frequency-specific manner, tACS is an unprecedented method of choice, since it can target and interfere with the specific intrinsic oscillations of the brain region (68).

There is a substantial variability of responses to NIBS techniques across subjects on an individual level (69). Beside the methodological factors of stimulation, which generally affect both inter- and intra-subject variability, there is a number of other determinants which have to be taken into account including anatomical features of the head and brain (70), initial level of brain function (71, 72), genetics (73), development, and aging (74).

### NIBS Studies on the Transition to Chronic Musculoskeletal Pain

The two most explored NIBS targets in pain are the primary motor cortex (M1) (19), which has been shown to undergo reorganization in chronic pain conditions (75), and the dorsolateral prefrontal cortex (DLPFC), due to its role in the affective and motivational components of pain (76). We discuss the contribution of NIBS studies targeting these two regions in the mechanistic understanding of chronic pain development. We then discuss additional NIBS targets that could potentially be beneficial to provide a mechanistic explanation of pain chronicity.

To uncover the mechanisms behind pain chronicity, the NIBS studies that are helpful are the ones that (1) focus on an experimental induction of prolonged pain in healthy individuals and follow the course of pain progression and pain resolution, thus providing a time course of pain development in relation to extended painful stimulation, or (2) focus on stimulation effects on clinical and induced pain in chronic pain patients to disentangle brain alterations from non-clinical compared to functionally and structurally altered clinical brain states, in relation to findings from (1).

#### Studies of Long-Term Pain Induced in Healthy Subjects

Through the experimental application and manipulation of pain in healthy individuals, we can monitor the time course when pain develops and gradually resolves and relate these changes with other clinical or neural measures. Prolonged pain in healthy humans can mimic symptoms seen in chronic conditions such as increased pain sensitivity (hyperalgesia), increased sensitivity to sensory stimuli (allodynia), or muscle soreness (77, 78).

Among the available NIBS studies of long-term pain induced in healthy subjects, NIBS has been used in the context of central sensitization, which has been proposed to underlie pain chronicity (79), and can manifest as secondary hyperalgesia or increased pain sensitivity at non-painful remote sites (80), and allodynia, painful sensation to usually non-noxious stimuli (81), shown in chronic back pain (82) and fibromyalgia (83). Available TMS studies applied both, stimulation protocols (rTMS) to modulate neural activity, and single pulse TMS as a measurement protocol to investigate cortical excitability changes and organization within the circuits of the motor cortex network.

##### Motor Cortex

Motor cortex was often targeted with NIBS due to analgesic effects that stimulation of this region has exhibited, most successfully when the target within M1 was the somatotopic representation of the painful body area (84).

Meeker et al. (85) delivered 1mA over the left M1 following the application of capsaicin on the right leg of 27 healthy subjects. They used capsaicin—heat pain model (C-HP model) where the thermode was attached to the participant's leg after the incubation of capsaicin applied into the bandage on the right leg. Warmth, heat, mechanical pain thresholds and suprathreshold mechanical pain ratings were obtained before the heat exposure, and the heat pain scores were assessed every minute throughout the heat exposure. Anodal tDCS started 12 min after the application of capsaicin and was delivered for 20 min. Additionally, extent and intensity of secondary mechanical hyperalgesia, and residual heat pain intensity were assessed at four time points, up to 65 min after removal of capsaicin and tDCS stimulation. In addition, 15 subjects from this study who have developed secondary mechanical hyperalgesia underwent three fMRI sessions before and after the application of the C-HP model receiving either anodal, cathodal, or sham tDCS in each session. Painful mechanical stimuli using weighted probes were assessed during and after the scanning sessions. Anodal tDCS renormalized the BOLD activation in several brain regions including mPFC, pregenual anterior cingulate cortex (pgACC), the periaqueductal gray (PAG), and brainstem, whose activity was prominent in response to mechanical pain, supporting the involvement of descending inhibitory circuits to supress prolonged influx of nociceptive stimuli (85). However, no effect of anodal stimulation was found on primary hyperalgesia (heat stimuli). This is consistent with a study that used repetitive heat stimuli in healthy individuals and found no effect of motor cortex tDCS stimulation on heat hyperalgesia (86). Meeker et al. (85) interpreted their findings as evidence that, due to its effects on secondary hyperalgesia, M1 likely influences supraspinal circuits that are altered due to central sensitization. The capsaicin injection was indeed shown to decrease regional cerebral blood flow (rCBF) in right the mPFC and increase rCBF in the caudal part of the right anterior cingulate cortex (ACC) after application of 1 Hz rTMS over right M1, as revealed by single-photon emission computed tomography (SPECT) (87). Moreover, the authors showed that pain decreased significantly after active rTMS compared to sham, and pain reduction significantly correlated with previously reported rCBF changes in mPFC and right ACC. This suggests that M1 is strongly related to mPFC and ACC regions during pain perception (87). In line with previous findings, Hughes et al. showed that compared to sham, active tDCS over M1 significantly reduced dynamical mechanical allodynia and mechanical pain sensitivity initiated by capsaicin-induced pain applied before tDCS in 12 healthy subjects. The authors concluded that M1 exhibits top-down modulation of inhibitory descending pathways to reduce the increased excitability in the dorsal horn, which has previously been associated with the development of allodynia (78).

In contrast to previous findings, (77) reported no significant effect of 10 Hz rTMS over the right M1 on motor excitability nor on the conditioned pain modulation (CPM), a reliable indicator of endogenous descending inhibitory pain control (88). Compared to a sham condition, active rTMS delivered over 5 successive days reduced the intensity of the pain induced by injection of neuronal growth factor (NGF) in the right forearm of 30 healthy participants. NGF induced pain spanning weeks, therefore more closely mimicking prolonged pain than capsaicin. Subjects in both groups developed multifocal, widespread pain, resembling the pattern seen in the chronic musculoskeletal pain conditions.

rTMS also reduced muscle soreness, narrowed the painful area, and increased pressure pain thresholds, and by day 14, the last experimental session, almost completely resolved muscle soreness and pain. Interestingly, rTMS did not exhibit significant effects on corticomotor excitability (activated cortical map volumes were increased over time in a similar fashion compared with the sham condition). Since the CPM task and motor excitability remained unaltered, the authors concluded that the observed effects are neither likely to be the result of M1 stimulation affecting descending pain inhibition networks, nor that they might emerge from local changes in M1. They rather discussed that the beneficial effects might have arose from changed activity of areas connected to M1 that are involved in pain processing or in affective processing of pain. Since these results were not confirmed by imaging, the mechanisms remained unclear. Nevertheless, it is plausible that M1 can indeed affect the activity of ACC, thalamus, insula, or DLPFC, as shown by imaging studies and by studies using electric field modeling to determine the current spread after M1 stimulation (89, 90).

Schabrun et al. (91) examined pain processing in an already sensitized system that resembles chronic conditions. They tracked M1 transient adaptation in response to saline injection in addition to NGF injection. The study involved 12 healthy subjects who were injected with NGF on day 0 and day 2, followed by an assessment of corticomotor excitability. Hypertonic saline was also injected on day 4 to enhance pain in an already sensitized system and measures of motor function and organization were assessed during induced pain lasting about 10 min, and again after the pain had resolved. Interestingly, TMS measurement protocol showed that motor cortex reorganization assessed by motor maps and number of discrete peaks in M1 activity occurred as early as on day 4 in response to the onset of pain and muscle soreness. Corticomotor excitability, assessed by MEP amplitude, was unchanged directly after the NGF injection, but increased on day 2 following repeated NGF injection in an already sensitized system (91). In contrast to what is known in chronic pain conditions, where the extent of M1 reorganization was associated with pain severity (75), this study showed that M1 reorganization was not associated with the development of pain severity and disability. This suggests that changes in muscles, rather than pain, are predominately driving early plasticity (91). Additionally, the authors argue that M1 reorganization is probably driven by a release of intracortical networks, as the observed increased intracortical facilitation enables redistribution of muscle activity from the affected site to non-affected surrounding areas. Disturbed balance between inhibitory and facilitatory motor circuits has been observed in various chronic pain conditions (92), but the results were not always straightforward.

Given that rapidly occurring neuroplastic changes often relate to pain duration in chronic pain conditions, it has been suggested that these changes could be preceding the chronic stage and therefore represent a risk factor for chronicity (75, 93). Moreover, individual differences in motor plasticity could underlie vulnerability to pain development, with some individuals adopting maladaptive changes due to abnormalities in pre-existing brain circuits characteristics. For example, Seminowicz et al. (94) showed that differences in motor cortex changes in response to NGF injection were not apparent when analyzed on the group level but emerged when individuals were divided according to their excitability responses. In individuals who showed corticomotor facilitation, motor maps were increased, whereas participants who showed depressed responses of corticomotor excitability, had reduced map volumes, and displayed higher pain severity and worse cognitive performance (94).

##### DLPFC

Among the studies that have addressed the DLPFC, Fierro et al. (95) showed how stimulation applied over the DLPFC affects motor cortex excitability during 1 h of capsaicin-induced heat pain. The authors first assessed how induced pain affects corticospinal excitability and short intercortical inhibition (SICI). As pain developed, reduced corticospinal excitability assessed by MEP amplitudes was reported together with intracortical disinhibition on the contralateral motor cortex, evidenced by SICI using paired-pulse TMS over M1. Interestingly, 5Hz rTMS over the left DLPFC delivered 10 min after capsaicin application reversed effects observed within the motor cortex, at the same time lowering pain ratings. The control condition designed to explore the effects of DLPFC stimulation on motor cortex excitability in absence of pain showed no effect upon motor cortex excitability. This suggests that pain might mediate the relationship between activation of the DLPFC and motor cortex and that DLPFC influences on pain might induce changes in the motor cortex. Moreover, such findings also show that DLPFC stimulation might be able to reverse excitability changes induced by pain (95). Importantly, motor excitability changes were associated with concurrent high pain ratings, but as motor cortex inhibition started to diminish, pain ratings were still high. These findings suggest that perceived pain intensity was at least partially independent of the observed changes in excitability of the motor cortex. This is in accordance with research that used infusion of hypertonic saline in healthy adults, which supressed motor evoked potentials immediately as pain reached the pain threshold, supressed up to 25 min after the pain declined. This could imply that recovery from acute pain itself does not prompt the brain to change accordingly, since the brain is not only shaped by the presence or absence of acute, but also by previous pain-related learning processes (96). This is in line with imaging evidence of shifted pain processing from nociceptive circuits to circuits involved in emotion and learning (5). It is thus conceivable that changed motor function is driving changes in motor organization, but motor reorganization itself is not a (sole) generator of chronic pain.

Studies that applied brain stimulation shortly after induced pain onset showed how a specific region can foster recovery from pain, but since stimulation was usually initiated when pain already developed, these studies cannot tell if such a targeted stimulation would also prevent the development of pain. To investigate whether stimulation applied before pain onset can induce early recovery, Seminowicz et al. (97) applied rTMS over left DLPFC before injecting NGF to the right forearm of 30 healthy subjects. The study protocol involved rTMS stimulation on 5 consecutive days beginning before first NGF injection that was applied in the initial experimental session and 2 days afterwards. Compared with sham TMS, active TMS significantly reduced pain severity, muscle soreness and the size of the painful body area compared to sham over time, while depression, anxiety, Positive and Negative Affect Schedule scores, and pain catastrophizing scores remained unchanged. There was a trend toward better performance on an attention task post-stimulation. The authors concluded that the mechanism of action was possibly either through descending modulatory endogenous circuits or by affecting cognitive aspects of pain (97). Effects of TMS were tested on each of the 5 days of intervention, and up to 14 days and were stronger on the intervention day and were detectable up to 3 days after the intervention, which might be indicative of immediate effects rather than initiation of long-term plasticity-like effects.

NIBS studies underline the importance of the DLPFC in pain chronicity, since it seems that DLPFC affects not only the affective but also sensory component of the pain, independently of motor cortex activation. This is in accordance with a study in fibromyalgia patients where tDCS over DLPFC modulated heat pain thresholds (98). In further support of this finding, Lin et al. showed that pain reduction after DLPFC stimulation was not related to M1 activity, but rather through direct thalamic inhibition (99). A naloxone injection interfered with the analgesic effects of M1 stimulation, while it had no effect on DLPFC stimulation, suggesting different mechanisms behind the effects on pain of these two cortical regions (100). In addition, anodal M1 tDCS and anodal DLPFC showed differential effects on other cortical and subcortical areas, as revealed by resting state fMRI pre and post tDCS stimulation (101).

Notably, structural and functional differences that may be “prewired” could make individuals more prone to the development of chronic pain. Lin et al. (99) found that immediate analgesic effects of anodal tDCS over left DLPFC are dependent on structural connectivity between left DLPFC and thalamus. Sham compared to tDCS over DLPFC stimulation revealed increased blood perfusion in posterior insula and thalamus on the left side and lower perfusion in M1, implying that these regions are involved in the processing of ongoing tonic pain, while anodal tDCS normalized this activity. Specifically, subjects who showed the strongest structural connectivity between left DPFC and thalamus, displayed the highest functional coupling between these two regions during anodal compared to sham tDCS (99). These findings are in line with previous research showing that resting state functional connectivity (102, 103), and individual morphology (104) predict pain sensitivity in healthy controls. Considering that pain sensitivity is a known risk factor for chronicity (105), these findings confirm the idea that structural and functional networks involving DLFPC relate to pain chronicity.

#### Brain Targets in Chronic Musculoskeletal Pain Patients

##### Motor Cortex

The processing of acute pain can be profoundly altered in chronic pain conditions, such that distinct patterns of alterations emerge across several brain regions including somatosensory cortex, thalamus, insula, motor and cingulate cortices (20, 106–108). The first study that explored how immediate effects of tDCS to M1 influence acute pain in chronic pain patients compared pain ratings to repetitive heat and electric stimuli in chronic low back patients before and after tDCS, with no significant outcome (109). In addition, although clinical pain was not the focus of this study, no effect on ongoing back pain emerged during or after tDCS compared to baseline or sham (109). Nevertheless, with improved tDCS parameters such as increased intensity and smaller, more focal electrodes, anodal tDCS over M1 was shown to decrease experimental pain scores in chronic low back patients (110). The peak value of current density was modeled to show that most of the current was delivered to M1, although one could not exclude that other regions such as DLPFC or primary somatosensory cortex (S1) may be affected. Interestingly, low back muscle activity did not show any differential response to stimulation (110). This is in line with the previous findings of prolonged pain in healthy subjects, where motor map volume remained unchanged after tDCS to M1, whereas pain decreased (111). These findings suggest that pain changes are not confined to motor cortex itself, but rather that M1 stimulation conveys its effect through other interconnected regions, or it acts in synergy with other regions.

To date, only one study employed non-invasive stimulation methods to study changes in the acute stage of clinical musculoskeletal pain. Chang et al. (112) recruited individuals experiencing acute low back pain lasting <4 weeks to elucidate which changes previously found in chronic stage of clinical back pain are present already early in acute clinical pain development. Employing electroencephalography (EEG), sensory evoked potentials (SEP) to non-painful electrical stimulation at the site of pain in area of the paraspinal muscles were recorded. Compared to healthy controls, corticospinal excitability assessed by TMS in M1 was lower in the 36 assessed patients. In addition, patients with low back pain had lower amplitudes of sensory evoked potentials related to secondary somatosensory cortex (S2) and ACC regions. Notably, the number of discrete motor map peaks did not show significant differences between subjects with and without pain, suggesting that the early phase of clinical pain development is not characterized by considerable motor cortex reorganization (112). However, this study was conducted in a cross-sectional manner. Further studies with follow-up measurements would be needed to examine whether the observed changes remained present up to the chronic stage.

##### Occipital Field

Occipital nerve field stimulation with subcutaneously implanted electrodes was shown to have positive effects in treating fibromyalgia (113). De Ridder and Vanneste (114) used source-localized resting-state EEG and tDCS over the occipital nerve field (OCF) to investigate mechanisms behind fibromyalgia pain. Using effective connectivity analyses, the authors showed that the connectivity changes between pgACC to the dorsal anterior cingulate cortex (dACC) were causally related to chronic pain. Specifically, active OCF tDCS compared to sham normalized disturbed effective connectivity from the pregenual anterior cingulate cortex to the dorsal anterior cingulate cortex, with a reduction of clinical pain. Considering the role of dACC in salience encoding and the role of pgACC in inhibitory pain control, the authors concluded that OCF tDCS exerted its modulatory effect via activation of the descending pain inhibitory pathway and de-activation of the salience network. Using directional functional connectivity measures to determine information transmission from one region to another it was revealed that pain increased with more information sent from dACC to pgACC, which led the authors to conclude that fibromyalgia is primarily driven by increased pain sensitization. Altered activity of pgACC, a part of the descending inhibitory system, is in accordance with imaging studies showing that smaller rostral ACC volume and cortical thickness in fibromyalgia patients were correlated with pain duration (106). The ACC is an important relay in the medial pain pathway and since it integrates sensory, attentional, and motivational components of pain, it could have a pivotal role in the development of chronic pain (115). Disrupted functional and structural connectivity between cingular areas and striatal regions have also been shown to be a predictor of the development of chronic pain (18).

##### Primary Somatosensory Cortex

rTMS and tDCS are the most prominent non-invasive techniques used in chronic pain studies. Oscillatory protocols such as tACS are largely unexplored and underrepresented in pain research (116). To date, only one study has examined tACS-related effects in chronic pain patients. Ahn et al. (117) administered 10 Hz tACS bilaterally over the S1 and showed reduction in ongoing back pain and disability ratings in chronic low back pain patients. In addition, this reduction correlated with increased alpha oscillations in the regions under the electrodes, but also within frontal areas, as documented by electrophysiological recordings (117). These findings suggest a causal relationship between somatosensory alpha oscillations and ongoing chronic pain and is in line with previous research that found associations between manipulations of neural activity at the alpha frequency in somatosensory cortices and the processing of phasic heat pain (118). However, the latter study showed that reduction in pain is dictated by the context of the painful stimuli, namely that tACS has an influence only when the intensity of the painful stimuli was uncertain. Moreover, electrophysiological studies indicated that the intensity of ongoing pain in chronic back pain patients is encoded by prefrontal gamma activity (119). This points to a prefrontal involvement in the early and subsequent evolvement of pain. These findings are consistent to the ones of Ahn et al. (117) since they found significant associations of pain severity not only with the somatosensory cortex, but also alpha oscillations in frontal regions. However, the question of the specificity of a certain region and its endogenous frequencies that give rise to the pain experience as it temporally unfolds, prompts further research that would combine stimulation and electrophysiological methods and utilize different types of pain. In accordance with what we know so far from imaging studies (5, 120, 121), it is conceivable that acute phasic pain is predominately processed by somatosensory cortices and subserved by its intrinsic frequencies. But when pain develops, a shift toward prefrontal areas was shown, and hence the longer lasting and chronic pain may primarily be governed by rhythmic activity in prefrontal regions (116). This does not exclude that activity in somatosensory regions might also change in response to chronic pain, as well as a possible interplay between alpha and gamma frequencies, known to engage in cross-frequency coupling and modulating each other (122).

## Future Directions

### Medial Prefrontal Cortex as an Additional NIBS Target

With respect to the non-invasive stimulation targets, only very recently additional targets such as the medial prefrontal cortex were investigated in chronic pain patients. In a study that attempted to modulate clinical pain manifestations via affective and attentional manipulations, chronic low back patients underwent active and sham HD-tDCS over the medial prefrontal cortex (123). Conditioned pain modulation was not altered by attentional and affective manipulations, and in addition HD-tDCS compared with sham did not show effects on the magnitude of the effects of these manipulations. However, as the authors acknowledged, the small sample size and the inclusion of only mild clinical pain might be the main reasons for the absence of an effect. Further studies with larger sample size and including severe clinical pain are needed to further our understanding of the role the mPFC in chronic musculoskeletal pain. Noteworthy, connectivity patterns of the mPFC were shown to be altered in chronic back pain (102, 124) and have been suggested as a predictor for pain chronicity (18, 30). A two-fold role of mPFC as a site exhibiting opposing effects on pain has also been suggested: it is a relay between higher and downstream areas in modulating pain perception, and its dysfunction can lead to chronicity via projections to striatal reward pathway that could lead to overstimulation of the thalamus and possibly of the insula (125). Due to the substantial body of mPFC neurotransmitters that tune the prefrontal processing of affective components of pain, mPFC has also been proposed as a central hub subserving cognitive and affective comorbidities seen in chronic pain states (126).

### NIBS Mechanistic Interference Framework

Studies that would be suited to mechanistically investigate the role of specific brain circuits in the development of chronic pain would have to consider several factors. Firstly, we could target neural activity previously found to be associated with and/or predictive for the development of musculoskeletal pain to investigate immediate effects of such a manipulation on pain regulation and demonstrate causal relation to chronicity. Studies thus far used either excitatory or inhibitory stimulation protocols depending on the method in question to potentiate analgesic effects, but rarely focused on up and downregulation with the aim to investigate a causal involvement of targeted area in pain processing. For instance, since it has been shown that activity in prefrontal brain regions in relation to spontaneous pain is increased in CBP patients (16), neuromodulation that inhibits prefrontal activity should decrease spontaneous clinical pain. In the same vein, if stimulated in an excitatory manner, prefrontal activity should amplify pre-existing overactivation and result in upregulation of pain intensity.

However, there are inherent challenges related to NIBS studies in chronic pain aiming to arrive to mechanistic explanations. A common non-invasive stimulation approach to investigate the causal role of a brain region in a specific behavioral or cognitive domain follows the “virtual lesion” principle. Hereby brief disruption of normal brain activity leads to immediate effects on the behavioral and/or cognitive level reflected by a changed response to the experimental task (51). Given that chronic pain is a subjective experience and thus is not experimentally induced, the task in this case is highly reliant on the subjects' perception and ability to transfer that perception into self-reported pain ratings. On the same grounds, chronic ongoing pain cannot be precisely time-locked to the stimulation as it can be for other experimental stimuli, such as, for example, visual stimuli delivered with millisecond precision concurrent with a stimulation pulse (127). In addition, stimulation procedures, particularly TMS, can themselves be painful depending on the site of the stimulation (128), hence they can interfere with the perception of ongoing clinical pain. Due to all these reasons, effects are investigated post-stimulation rather than in an online fashion, which, however, imposes time delay and make effects more indirect (129). If explored online, i.e., during the stimulation, caution should be taken when interpreting the effects on chronic ongoing pain.

Next, careful consideration of the control conditions is a prerequisite toward more conclusive mechanistic interference. Sham procedures ensure the control of non-specific effects of TMS such as placebo effect, auditory noise, or sensory percepts of pulse discharge. To evaluate specificity of the brain region, TMS can be applied over another area presumably not involved in pain processing (129). To confirm behavioral specificity, effects of the targeted area should be confined to the task in question (130), in this case to the (pre)chronic ongoing pain. This could be examined by introducing a control task that requires neural processes that are not involved in pain perception, such as, for example, visuo-motor coordination (16). The power of mechanistic evidence increases with increasing control conditions (130), but also decreases statistical power. There is therefore a compromise to be found between the choice of control conditions and the conclusive mechanistic interference.

Importantly, our recent tACS studies emphasize the need for an active control condition to explicitly test frequency specificity, which is usually ignored in most NIBS studies to date (131, 132). NIBS studies should follow the general recommendations in terms of good scientific practice for planning a tACS experiment, which include the recommendation to choose an appropriate control frequency to demonstrate frequency specificity (54, 133). Following this rationale, the optimal control condition would be a frequency at which no modulatory effect would be expected. Therefore, it is important to avoid a synchronization between the frequency of interest and the control frequency.

Precise targeting is necessary to restrict stimulation effects to the desired region. Due to intrasubject variability, MRI-based TMS neuronavigation should be preferred instead of the traditional 10–20 EEG system positioning that is less accurate and has been found to induce different electrical field distributions compared to imaging-based localization (134, 135).

Next, to track which changes are pre-existing in the chronic stage and at the same time putative causes of chronicity, NIBS interventions might be introduced in the acute and then to and in the subacute stage. In this manner, any brain activity-pain relation found before the chronic stage would be marked as a potential risk factor for pain chronicity. In contrast to brain imaging studies which enable mainly correlational evidence between brain activity and pain, NIBS would provide controlled manipulation cause-effect relationships.

### Combining NIBS With Imaging and Electrophysiological Methods

The mapping of NIBS-related effects could be performed by combining NIBS and imaging or NIBS and electrophysiological methods. Non-invasive stimulation is primarily confined to the superficial cortical layers (38), but via interconnected areas it can have an effect on subcortical regions. Therefore, the stimulation effect of the target could be fully prescribed to the activation of deeper layers (40). Due to these reasons, more conceivable and more precise mechanistic explanations would require imaging the effects of the induced stimulation immediately after the intervention to obtain a clearer picture of the affected circuits. Previous studies showed that tACS can induce BOLD changes even in the absence of behavioral modulations (136–138). Different stimulation frequencies can lead to both an increase and a decrease of brain activity. Moreover, the frequency range in which the change in brain activity occurs can coincide with the stimulation frequency, or lie in a different frequency range (131, 139).

Since it is conceivable that complex perception such as the pain experience depends on several key factors, the neuronal network on a whole, rather than an isolated brain region, is highly likely to be affected as pain progresses to the chronic state. One specific characteristic of the neural network is its oscillatory activity (140), thus its exploration could aid our understanding of the role of brain networks in the transition to chronic pain. From the pool of non-invasive stimulation methods, rTMS and tACS emerge as approaches able to influence intrinsic rhythmic activity via the proposed mechanism of entrainment (68). This concept refers to synchronization of the rhythmic activity of a physical system to an external periodic oscillator (141), and in case of neural endogenous oscillations reflects their coupling to the stimulator (67). rTMS and tACS can act in a frequency-specific manner (68), and thus a causal role of brain oscillations and regions that recruit them could be investigated in (pre)-chronic pain patients. Electrophysiological research showed an association between prefrontal gamma activity and ongoing back pain intensity, indicating therefore that the prefrontal areas are vital parts of long-lasting pain development, showing that medial prefrontal cortex encodes tonic pain at the gamma frequency in healthy controls (142), and changes in prefrontal gamma activity are associated with changes in ongoing back pain intensity (119). It remains however unknown if additional oscillation frequencies could be involved in pain chronicity, and this deserves further investigation.

To date, only one study in chronic musculoskeletal pain patients at our knowledge, combined tACS and EEG to successfully demonstrate an impact on chronic low back pain via an influence on somatosensory alpha frequencies (117). Network pathology in chronic musculoskeletal pain should be further investigated because for instance, cross-frequency coupling could subserve interactions between large-scale neural networks and local dynamics (122). If applied in a longitudinal framework, such research could, in addition, reveal whether the communication within constituents of brain networks is affected in the states preceding chronic pain development, or at which time point such alterations possibly emerge.

## Concluding Remarks and Implications for Clinical Practice

Overall, there is a great need to employ the NIBS interference framework to elucidate changes in brain circuits as potential causal factors of the development of chronic musculoskeletal pain. This research should be built upon previously demonstrated significant predictors of chronicity in imaging studies that provide potential targets of non-invasive stimulation. Non-invasive stimulation applied in models of prolonged pain or in chronic musculoskeletal pain patients thus far seems to confirm the importance of prefrontal regions in the transition to chronic pain. Importantly, NIBS showed that interventions applied preceding or in an early time windows of long-lasting pain can revert maladaptive responses. Moreover, NIBS findings point to the relevance of the connectivity patterns and deeper areas, justifying their targeting by methods such as neurofeedback (143). Additionally, it highlights fast plastic changes in the motor cortex in response to pain onset, alongside interindividual differences, which calls for more investigation. Here, studies in other pain conditions may also provide important information. The results for chronic musculoskeletal pain are mirrored in studies on neuropathic pain suggesting that there might be considerable overlap in the brain processes between the two types of pain. This has been demonstrated, for example, very recently in a study by Attal et al. (144), where it was shown that M1-rTMS, but not DLPFC-rTMS, induces significant effects on pain intensity changes compared to sham-rTMS.

Furthermore, an intact descending inhibitory pathway seems necessary to counteract early maladaptive changes associated with central sensitization, although this could be related to the predominance of central sensitization in the mechanism behind symptoms, since not all chronic pain patients exhibit CS symptoms (145). It has been demonstrated that chronic conditions with absence of any tissue injury exhibit less efficient descending pain modulatory system as assessed by the CPM paradigm (146). At the same time, symptoms of central sensitization are not necessarily alone good predictors of chronicity, but rather work jointly with other factors such as psychological determinants, as shown in an acute stage of low back pain using the CPM paradigm and pain thresholds (147). Nevertheless, pain management directed at restoring functionality of descending inhibitory pathways in an early manifestation of the central sensitization phenomena could have important implications for chronic pain patients that exhibit those symptoms. Previous studies indicate that state-triggered and closed loop stimulation boosts effects of non-invasive transcranial brain stimulation [for review, see (148)].

Employment of novel variants of non-invasive stimulation, such as theta burst stimulation that has a potential for more reliable excitatory and inhibitory effects on brain regions (149), should be encouraged in an interference driven approach. This method successfully ameliorated pain in several other chronic pain conditions such as chronic orofacial pain, complex regional pain syndrome, and central neuropathic pain (150–152) and thus incites therapeutic applications. Another recent NIBS approach, a form of tACS called transcranial random noise stimulation (tRNS), has proven efficient to consistently induce increased cortical excitability with effects lasting up to 1 h after the stimulation (153). tRNS uses the alternating electric current following a random white noise spectrum (54). In addition, stimulation in high frequency range (80–250 Hz) was shown to be a potent stimulation protocol to increase human cortical excitability during and after the end of stimulation (154), opening a possibility to explore such a protocol in pathological conditions. A multi-coil magnetic stimulation design shown to modulate anterior cingulate cortex in fibromyalgia patients could be one possibility to effectively target deeper areas (155).

Last, spinal cord stimulation (SCS), as another well-established therapeutic option for the treatment of chronic pain, has also been examined for patients with chronic back pain, specifically those with failed back syndrome (156). A functional imaging study in patients with chronic pain showed that SCS reduced pain sensation along with abnormal functional connectivity between somatosensory and limbic circuits and increased the connectivity between somatosensory areas and the default mode network (157). These data also point to a close interaction of sensory and emotional processing networks in chronic pain that could be targeted in treatment.

A mechanistic understanding of the transition from acute to chronic musculoskeletal pain is needed to permit targeted early intervention (158). However, our knowledge of chronic pain mechanisms is still limited and the evidence for mechanistically guided treatments is sparse.

## Author Contributions

MK, HF, and FN conceived the design. MK drafted the manuscript. MK, VM, JA, HF, and FN edited the manuscript, provided the content, and contributed to the organization. All authors approved the submitted version.

## Conflict of Interest

The authors declare that the research was conducted in the absence of any commercial or financial relationships that could be construed as a potential conflict of interest.

## Publisher's Note

All claims expressed in this article are solely those of the authors and do not necessarily represent those of their affiliated organizations, or those of the publisher, the editors and the reviewers. Any product that may be evaluated in this article, or claim that may be made by its manufacturer, is not guaranteed or endorsed by the publisher.
